# Using Electronic Health Records to Mitigate Workplace Burnout Among Clinicians During the COVID-19 Pandemic: Field Study in Iran

**DOI:** 10.2196/28497

**Published:** 2021-06-03

**Authors:** Pouyan Esmaeilzadeh, Tala Mirzaei

**Affiliations:** 1 Department of Information Systems and Business Analytics College of Business Florida International University Miami, FL United States

**Keywords:** COVID-19, pandemic, clinician burnout, electronic health record, health information technologies, hospital intervention

## Abstract

**Background:**

The COVID-19 pandemic spread worldwide in 2020. Notably, in the countries dealing with massive casualties, clinicians have worked in new conditions characterized by a heavy workload and a high risk of being infected. The issue of clinician burnout during the pandemic has attracted considerable attention in health care research. Electronic health records (EHRs) provide health care workers with several features to meet a health system’s clinical needs.

**Objective:**

We aim to examine how the use of EHR features affects the burnout of clinicians working in hospitals that have special wards for confirmed COVID-19 cases.

**Methods:**

Using an online survey, we collected data from 368 physicians, physician assistants, and nurses working in six hospitals that have implemented EHRs in the city of Tehran in Iran. We used logistic regression to assess the association between burnout and awareness of EHR features, EHR system usability, concerns about COVID-19, technology solutions, hospital technology interventions, hospital preparedness, and professional efficacy adjusted for demographic and practice characteristics.

**Results:**

The primary outcome of our study was self-reported burnout during the COVID-19 pandemic. Of the 368 respondents, 36% (n=134) reported having at least one symptom of burnout. Participants indicated that the leading cause of EHR-related stress is inadequate training for using technology (n=159, 43%), followed by having less face-to-face time with patients (n=140, 38%). Positive perceptions about the EHR’s ease of use were associated with lower odds of burnout symptoms. More interventions, such as clear communication of regulations; transparency in policies, expectations, and goals regarding the use of technology in the clinical workflow; and hospital preparedness to cope with the challenges of the pandemic, were associated with lower odds of burnout.

**Conclusions:**

The use of EHR applications, hospital pandemic preparation programs, and transparent technology-related policies and procedures throughout the epidemic can be substantial mitigators of technology-based stress and clinician burnout. Hospitals will then be better positioned to devise or modify technology-related policies and procedures to support physicians’ and nurses’ well-being during the COVID-19 pandemic. Training programs, transparency in communications of regulations, and developing a clear channel for informing clinicians of changes in policies may help reduce burnout symptoms among physicians and nurses during a pandemic. Providing easily accessible mentorship through teleconsultation and 24-hour available information technology support may also help to mitigate the odds of burnout.

## Introduction

Burnout has attracted more attention in health care since it is considered a trigger for health care professionals’ physical and mental problems [1]. Clinicians’ burnout may negatively affect the quality of care, cost of health care delivery, productivity, and patient satisfaction [2]. Prior research indicates different organizational, relational, and work-related factors contributing to clinicians’ burnout [3,4]. COVID-19 was first identified in Wuhan, China and has since become a global pandemic that has spread to more than 150 countries and affected over 9.6 million people worldwide [5]. Recently with the spread of the COVID-19 pandemic, burnout syndrome and physical exhaustion among clinicians have become even more pronounced. Declaring the COVID-19 outbreak as a pandemic by the World Health Organization has raised concerns about its possible detrimental effects on clinicians’ workload and well-being [6].

During the outbreak, clinicians have been exposed to patients with severe or mild symptoms. Since respiratory droplets and close contact are the main transmitters of COVID-19, clinicians are at higher risk of being affected [7]. Besides the health-related stress, health care centers have had a sudden increase in phone calls, patient portal messages, appointment requests, ambulatory care visits, and walk-in patients [8]. Accommodating patients with lots of concerns, questions, and clinical demands has also increased health care professionals’ workload. These burnout symptoms are exhibited by not only health care professionals who are specialized in infectious diseases but also physicians with different specialties, and nurses may experience more significant work pressure dealing with suspected and confirmed cases [7]. Previous studies have provided evidence to indicate that clinicians have experienced stress, anxiety, and depression during the COVID-19 pandemic [9,10].

Research to date has identified several contributing factors associated with burnout, including health information technology (HIT). Electronic health record (EHR) systems, for example, are often seen as cumbersome to use, failing to fulfill the promise of improved health care delivery and little more than a means of meeting regulatory and billing requirements [11,12]. However, during the COVID-19 outbreak, health care organizations are establishing different strategies and leveraging several health information technologies to improve pandemic management [13]. One of the technology-based tools that can support the standard management of patients during the current pandemic is the EHR [14,15].

The EHR has some features to enable the use of standardized processes such as scripted triaging, immediate health information exchange, real-time data analytics, electronic check-in, self-screening pages, and telemedicine [16]. Providing remote care through telemedicine and teleconsulting for patients became more prevalent during the outbreak. Using patient portals to provide instructions and medication prescriptions increased as well. There remains considerable debate in the informatics community about the actual role health information technologies play in shaping clinician burnout during the pandemic. Existing research suggests that technologies may be confounded with other important causes, including changes in regulatory mandates, increased clinical volumes, and flexibility to mitigate ad hoc challenges. Regardless of the role information technology (IT) plays in clinician burnout, innovative solutions to alleviate burnout during the pandemic are urgently needed. A recent article explains that “EHR innovations cannot help to mitigate clinician burnout without careful consideration of the socioecological context in which these innovations occur, including organizational culture, the healthcare marketplace, technology ecosystem, and national policy” [17]. There is a lack of research investigating the impact of this pandemic on burnout among clinicians in resource-limited countries and the possible impact of HIT on clinicians’ stress and burnout [18].

We investigate how clinicians’ awareness of EHR features and perception of EHR system usability as well as their perceptions about the hospital policies and preparedness can help reduce the burnout caused during the pandemic for clinicians in Iran, which was one of the resource-limited countries that had been reported to be among the first 15 countries to have COVID-19 patients and deaths [19]. As of April 8, 2021, Iran has 1,984,348 total confirmed cases and 63,699 deaths [19]. EHRs are still developing in Iran, and various hospitals are at a different level of adoption and use of EHRs. Hospitals in Iran use several EHR applications and informatics infrastructure for outbreak management [14,20]. However, little is known about how the use of EHR capabilities and clinicians’ perceptions of this technology in the Iranian health system during the COVID-19 outbreak successfully reverse burnout among clinicians to manage this novel infection. This study’s findings can contribute insights to develop better strategies for using EHRs, refining policies, and enhancing preparedness to reduce the increasing rate of burnout symptoms during the pandemics in resource-limited countries.

## Methods

### Setting

There are 118 hospitals in Tehran, the capital city of Iran, and among them, only 15 hospitals have wards specialized for quarantining and treating patients with COVID-19 (at the time of this research). In this study, we considered six of these hospitals with large administrations. These six hospitals were mainly selected because they were among the first hospitals in Tehran that have been quarantining and treating patients with COVID-19. They encompass outpatient primary and specialty medical and surgical care as well as emergency patient care. These hospitals were pioneer health systems in the country to care for COVID-19–positive patients. They created a special ward for COVID-19–infected patients in February 2020. They served as a quarantine site for confirmed cases and established a center to monitor and adapt to rapidly changing conditions and recommendations from local, state, and federal officials. The hospital characteristics are summarized in Table 1. These hospitals have implemented one of the main commercially available EHR systems (brands) in Iran with similar applications, functionalities, and features. Some of these vendors provide services to public hospitals, and some offer services to private hospitals. All EHR systems offer some of the basic features such as viewing laboratory results, collecting patient demographics, listing patient problems, and highlighting out-of-range laboratory results. However, other advanced features such as sending and receiving reminders and messages and remote access to health information have been scarcely or not implemented yet. Table 2 shows the prevalence of EHR features implemented in the hospitals of interest in this study.

**Table 1:** Hospital details.

Type of hospital	Physicians, n	Nurses, n	Outpatients in a year, n	Total patients (annually), n	Beds, n
Private hospital	150	185	16,000	23,000	50
Private hospital	90	170	15,000	20,000	45
Private hospital	70	180	15,000	22,000	50
Public hospital	250	400	11,000	15,000	330
Private hospital	180	320	20,000	31,000	445
Private hospital	120	400	24,000	30,000	200

**Table 2:** Implementation of EHR features among the hospitals in this study.

EHR^a^ feature	Hospitals (N=6), n
View laboratory results electronically	6
Collect patient demographics electronically	6
List patient problems electronically	6
Highlight out-of-range laboratory results	6
List patient medications electronically	5
Create medical history and follow-up notes	5
Order laboratory tests electronically	5
Order radiology tests electronically	5
View electronic clinical notes electronically	4
View imaging results electronically	4
View allergy lists	4
Warn of drug interactions and contraindications	3
Personal human resources (payroll, benefits, training)	3
Preregister systems	3
Send prescriptions electronically to a pharmacy	2
View immunization records	1
Send medical information securely to other health care professionals	1
Receive reminders for guideline-based interventions	1
Receive reminders for preventive screening tests	1
Sending electronic referrals	1
Receive reminders for screens of chronic disease management	0
Sending electronic clinical messaging to patients	0
Remote access to EHR	0

^a^EHR: electronic health record.

### Research Design

This study was reviewed and approved by the involved hospitals. Data collection was conducted in the six hospitals using an online survey during May and August 2020. For data collection, we could not reach out to all physicians and nurses working in the six hospitals for two reasons. First, due to the COVID-19 pandemic, a large number of clinicians did not work in the same shifts as they worked before the outbreak. Second, many physicians and nurses of the hospitals did not work at the COVID-19 specialized wards. Therefore, our clinician population size was shortened to clinicians working in different shifts at the COVID-19–dedicated wards. Based on the information provided by the six hospitals’ administrations, the total population size was 586 clinicians. We asked the administrators of the COVID-19 specialized wards to distribute the survey online among all clinicians working in different shifts at the COVID-19 wards during May and August 2020 (4 months).

Since WhatsApp is a popular communication app in Iran, the survey was mainly administered via WhatsApp to physicians (with different specialties) and nurses who were directly exposed to patients who were suspected of or infected with COVID-19. The survey was designed to measure facets of HIT use, including EHR functionality, electronic prescribing, health information exchange, other technology-based tools, and informatics applications.

Survey questions also examined the effects of using HIT on workflow, patient care, and clinician burnout during the COVID-19 pandemic. The survey was first prepared in English; then, it was translated to Farsi by one of the authors. The survey was reviewed by a physician working in Iran, and based on the feedback, some modifications were made for clarity purposes. Next, the survey was translated back to English by a university professor in Iran to ensure the correct terms were used and the questions were clear and understandable to participants. Before data collection, survey questions were reviewed, evaluated, and approved by the IT departments and hospital administrations. Finally, the survey was conducted anonymously at the individual clinician level. The survey questions are provided in Multimedia Appendix 1.

In this study, the dependent variable was clinician burnout. We used a single item to measure clinician burnout. This single question was adapted from the physician’s work–life study [21,22]. The measure has six answers: (1) no symptoms of burnout, (2) under stress but do not feel burned out, (3) having one or more symptoms of burnout such as emotional exhaustion, (4) thinking about work frustrations as symptoms of burnout will not go away, (5) feeling completely burned out and seeking help, and (6) completely burned out and getting help now [23]. Following previous studies [18], we dichotomized this measure into “no symptoms of burnout” (≤2 on the 5-point scale) and “one or more symptoms of burnout” (≥3 on the 5-point scale). This single-item measure was validated for clinicians and exhibited a better sensitivity and specificity compared with the Maslach Burnout Inventory survey [24].

Next, we were interested in identifying the factors that influence burnout during the COVID-19 pandemic. Indicators of burnout include awareness of EHR features, EHR system usability, concern about COVID-19, use of technology solutions, use of hospital technology interventions, hospital preparedness, and professional efficacy. Table 3 shows the description of each indicator.

**Table 3:** Description and source of variables used in this study.

Measures	Description	Sources
Awareness of EHR^a^ features	The extent to which participants are aware of available EHR features	[25]
EHR system usability	Adapted from the System Usability Scale: giving a global view of subjective assessments of EHR system usability	[26]
Concerned about COVID-19	The level of concern of the participants about the effect of the COVID-19 pandemic in their life	[7]
Use of technology solutions	The extent to which individuals use technology solutions for coping with work-related stress	[27,28]
Use of hospital technology interventions	The extent to which individuals agree with the use of different organizational interventions at their workplace (hospital)	[29,30]
Perceived hospital preparedness	The extent to which clinician perceives the hospital is prepared to deal with the COVID-19 crisis	[31]
Professional efficacy	Adapted from Maslach Burnout Inventory-General Survey: the extent to which an individual’s perceived effectiveness and accomplishment at work	[32]

^a^EHR: electronic health record.

We included several control variables such as patients’ demographics and practice setting. Respondents provided information about their age, gender, marital status, years of practice, role, specialty, area of work, and hospital setting.

There is considerable debate in the health informatics community about the actual role health information technologies play in clinician burnout [1]. We are interested in identifying the specific factors related to EHRs that may cause stress among clinicians. Further, once we understand the causes of burnout, we are left to ponder innovative solutions to prevent or mitigate burnout. Existing research suggests that some technological advances may help to reduce burnout. However, more studies need to investigate the confounding effect of regulatory mandates and interventions that aim at improving communication, changing workflow, or addressing clinician concerns via quality improvement projects [33]. Therefore, we added additional questions in the survey to understand EHR-related causes of stress, other technology-based solutions, and policy interventions that may help clinicians reduce burnout. Respondents were asked to report stressors associated with the use of EHRs on a 5-point scale. The factors causing stress as a result of using EHRs were increasing computerization of practice, too much time spent on EHRs at work, spending an enormous amount of time on the EHRs at home, insufficient time for documentation, too much data entry, having less face-to-face time for conversation and examination with patients, and inadequate technology-related training for using EHRs at work. These measures were adopted from previous studies [23].

Using a 5-point scale, participants were asked to indicate to what extent using technology-based solutions can help them cope with work-related stress. The solutions were availability of training programs; availability of responsive IT support; possibility for telecommuting (working from home); using technology and tools to follow up with patients remotely (telemedicine); use of mobile apps for meditation, breathing, and relaxation; availability of help desk services; mentorship programs through teleconsultation; and communication groups via messaging apps (eg, WhatsApp, Telegram, and Viber).

Using a 5-point scale, we asked respondents to evaluate the effectiveness of the hospital’s policies, strategies, and interventions regarding the implementation and use of technology-based tools and informatics infrastructure at the workplace. The policies and interventions were developing standards for order entry and reporting among hospitals; using data analysts (specialists) to analyze patient data and elaborate on the patterns; designing an online survey to regularly measure satisfaction with technology and possible technology-related risks and stress; establishing a regular assessment to evaluate the effectiveness of technology in hospitals; using a systematic way to measure workplace burnout and analyze the results; formulating transparent policies, expectations, and goals regarding the use of technology in the clinical workflow; applying clear regulations about the responsibility of clinicians when medical errors occur using technology; and providing incentives for using technology meaningfully for health care delivery. These items were followed by an open box for the participants to add effective interventions or strategies.

### Statistical Analysis

We used SPSS Statistics V21.0 (IBM Corp) to conduct all statistical analyses. We used univariable statistics to describe the respondents’ demographic characteristics, the prevalence of burnout, general causes of stress at the workplace, EHR-related stress, and technology solutions to cope with stress. We used multivariable logistic regression to measure the association between burnout and measures of respondents’ awareness of available EHR features, perceived EHR system usability, level of concerns about COVID-19, use of technology solutions for coping with stress, use of technology at the workplace, hospital preparedness for the pandemic, and perceived professional efficacy. We controlled the model for respondents’ age, gender, marital status, role, area of work, specialty, and the number of years they have been in practice to ensure that these factors did not create bias in the results. We performed a sensitivity analysis using an ordered logit model to examine whether the dependent variable (burnout) was represented by its ordinal response categories instead of the dichotomized response categories included in the primary analysis. We also tested the burnout association with the number of patients with COVID-19 that have been cared for by the respondents to identify whether more patients are independently associated with burnout. The results of the sensitivity analysis are reported in Multimedia Appendix 2. Further, to ensure the robustness of the coefficients, we repeated the analysis by considering within-hospital correlations and controlling for the hospital effect. Results are reported in Multimedia Appendix 3 and show that all coefficients remain robust.

## Results

Due to a large amount of abrupt tension and irregularity caused by the pandemic, only 373 clinicians initially attempted the survey. After removing incomplete answers, we finally collected 368 fully completed surveys that included 147 nurses, 161 physicians, and 60 physician assistants providing care for patients with COVID-19 (Table 4). The majority of the respondents were female (n=266, 72%), and 68% (n=252) were married. Among the respondents, 22% (n=80) were younger than 35 years, 42% (n=154) were aged between 35 and 44 years, 21% (n=78) were aged between 45 and 54 years, and 16% (n=56) were 55 years or older. There was about an equal number of respondents working in emergency departments, intensive care, and operating rooms (all n=67, 18%), while 34% (n=124) reported working in other inpatient services, and 12% (n=43) reported working in outpatient services. About a quarter of the respondents reported having less than 5 years of experience in practice, while 20% (n=73) reported between 6 to 10 years of experience, 19% (n=71) reported between 11 to 15 years of experience, 22% (n=82) between 16 and 20 years of experience, and 13% (n=46) reported having more than 20 years of experience in practice. The majority of the respondents were from private hospitals (n=310, 84%). About 43% (n=159) of the respondents reported having at least one extra night shift per week since the beginning of the pandemic.

**Table 4:** Characteristics of respondents.

Characteristics			Sample (N=368), n (%)
**Age (years)**			
	<35	80 (22)	
	35-44	154 (42)	
	45-54	78 (21)	
	55-64	35 (10)	
	≥65	21 (6)	
**Gender**			
	Male	102 (28)	
	Female	266 (72)	
**Marital status**			
	Married	252 (68)	
	Single	116 (32)	
**Role**			
	Nurse	147 (40)	
	Physician	161 (44)	
	Physician assistant	60 (16)	
**Area of work**			
	Emergency department	67 (18)	
	Intensive care unit	67 (18)	
	Other inpatient services	124 (34)	
	Outpatient services	43 (12)	
	Operating rooms	67 (18)	
**Specialty**			
	Emergency medicine	27 (7)	
	Family medicine	38 (10)	
	Surgery	56 (16)	
	Anesthesiology	67 (18)	
	Gynecology	50 (14)	
	Nursing	56 (14)	
**Years in practice**			
	<1	3 (0.01)	
	1-5	96 (26)	
	6-10	73 (20)	
	11-15	71 (19)	
	16-20	82 (22)	
	>20	46 (13)	
**Hospital setting**			
	Public hospital	58 (16)	
	Private hospital	310 (84)	
**Burnout prevalence**			
	I *enjoy* my work. I have no symptoms of burnout.	115 (31)	
	I am under *stress* and don’t always have as much energy as I did, but I don’t feel burned out.	119 (32)	
	I am *definitely burning out* and have one or more symptoms of burnout (eg, emotional exhaustion).	66 (18)	
	The symptoms of burnout I am experiencing won’t go away. I think about work *frustrations* a lot.	24 (7)	
	I feel completely burned out. I am at the point where I may need to *seek help*.	28 (8)	
	I am completely burned out, and *I am getting help*.	16 (4)	

**Figure 1:**
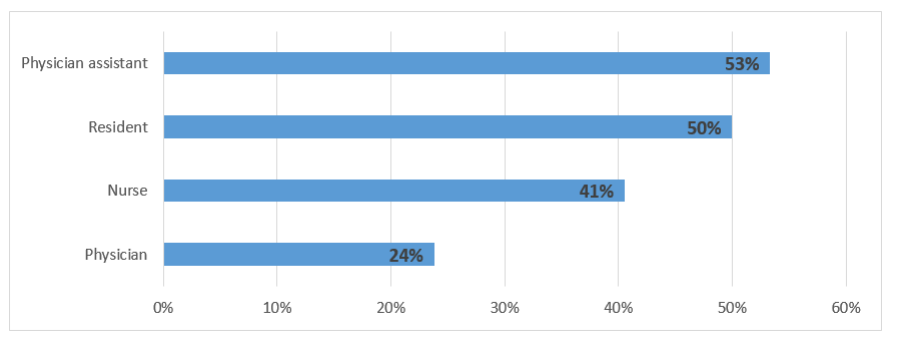
Percent of respondents reporting at least one symptom of burnout in their role.

A total of 36% (134/368) of the respondents reported having at least one symptom of burnout. The highest level of burnout was reported among the physician assistants (32/60, 53%), followed by nurses (Figure 1).

**Figure 2:**
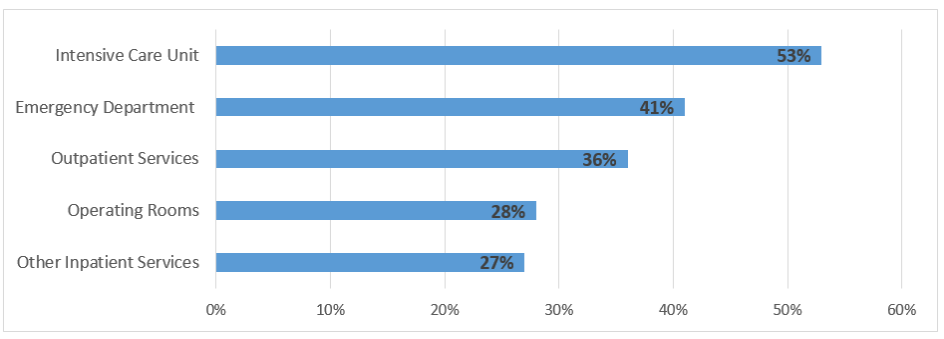
Percent of respondents reporting at least one symptom of burnout by area of work.

Regarding the area of work, most burnout symptoms were reported in intensive care units, followed by emergency department and other outpatient services (Figure 2).

**Figure 3:**
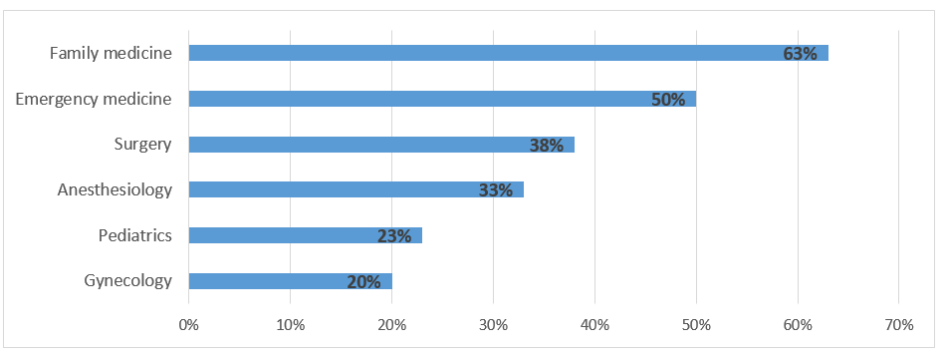
Percent of respondents reporting at least one symptom of burnout by specialty.

The specialties who experienced the most from burnout practiced family medicine and emergency medicine, followed by surgery and anesthesiology (Figure 3). Results show that family medicine physicians experienced the most from burnout. This finding is in line with several previous studies [34]. Family medicine physicians usually feel the most stress because they need to provide comprehensive health care for people of all ages. During the pandemic, family medicine visited more patients with similar symptoms (eg, common cold or flu). At the time of our study, people who felt that they might be at risk of being COVID-19 positive usually visited their trusted family medicine first for a check-up or advice. This also has some cultural reasons because family doctors are generally considered the most trusted physicians for families and the first choice to visit in case of an ailment. Thus, family medicine physicians visited various patients across all ages, genders, and diseases, and they felt the most stress and frustration under the pandemic situation.

Before running the logistic regression model, we assessed the reliability of the proposed measures. Table 5 presents some indicators and descriptive statistics for each measure. We assessed the reliability using Cronbach alpha for all measures. The recommended threshold value is above .70 [35], which implies adequate reliability for all measures in this study. For awareness of EHR features, since it was measured as a set of dichotomous items, we separately calculated an aggregate score adjusted for each hospital and used that score in the logistic regression model to assess its impact on burnout. Higher scores show a higher level of awareness about the available EHR features.

**Table 5:** Summary of the properties of the measurements.

Measures	Measurement type	Mean (SD)	Cronbach α
EHR^a^ system usability	10-item scale measured on a 5-point Likert scale	3.20 (0.71)	.85
Concerned about COVID-19	4 items measured on 5-point Likert scale	4.43 (0.67)	.72
Use of technology solutions	8 items measured on a 5-point Likert scale	3.12 (0.80)	.81
Use of hospital technology interventions	8 items measured on a 5-point Likert scale	2.89 (0.85)	.86
Hospital preparedness	5 items measured on a 5-point Likert scale	3.50 (0.86)	.87
Professional efficacy	4 items measured on a 5-point Likert scale	3.75 (0.79)	.88

^a^EHR: electronic health record.

Next, based on the multivariable logistic regression model while controlling for age, gender, marital status, role, area of work, specialty, years of experience, and type of hospital (Table 6), we identified that perception of EHR system usability, level of concern about COVID-19, hospital technology-related interventions, and level of hospital preparedness to cope with the pandemic are the significant factors associated with burnout among the clinicians. Positive perceptions about the ease of use of EHRs and a high level of confidence in understanding its function were associated with lower odds of burnout symptoms (OR –0.16, 95% CI –0.24 to –0.09; *P*<.001). In other words, the nurses and physicians who find EHRs useable and are confident in their knowledge and ability to work with it have lower odds of having any burnout symptoms compared to those who find EHRs difficult and cumbersome to use. Similarly, the more use of technology interventions implemented in the hospitals was associated with lower odds of burnout (OR –0.38, 95% CI –0.51 to –0.26; *P*<.001).

**Table 6:** The estimate of the association between demographic, practice, and EHR characteristics and 1 or more symptoms of burnout.

Variable			OR^a^ (95% CI)		SE		*Z* value		*P* value
Awareness of EHR^b^ features			0.03 (–0.07 to 0.13)		0.05		0.54		.59
EHR system usability			–0.16 (–0.24 to –0.09)		0.04		–4.16		<.001
Concerned about COVID-19			0.29 (0.11 to 0.48)		0.09		3.05		.002
Use of technology solutions			–0.06 (–0.14 to 0.03)		0.04		–1.31		.19
Use of hospital technology interventions			–0.38 (–0.51 to –0.26)		0.06		–5.95		<.001
Hospital preparedness			–0.23 (–0.38 to –0.09)		0.07		–3.05		.002
Professional efficacy			–0.06 (–0.23 to 0.11)		0.09		–0.70		.49
**Age (years; reference group: ≥65 years)**									
	<35	–0.11 (–3.48 to 3.09)		1.65		–0.07		.95	
	35-44	–2.13 (–5.49 to 0.79)		1.58		–1.35		.18	
	45-54	–0.88 (–4.05 to 1.98)		1.51		–0.59		.56	
	55-64	1.61 (–1.35 to 4.73)		1.51		1.06		.29	
**Gender (reference group: female)**									
	Male	–0.75 (–1.98 to 0.44)		0.61		–1.22		.22	
**Marital status (reference group: single)**									
	Married	–0.30 (–1.35 to 0.72)		0.53		–0.57		.57	
**Role (reference group: physician assistant)**									
	Nurse	–0.93 (–2.24 to 0.34)		0.66		–1.42		.16	
	Physician	–2.34 (–3.96 to –0.86)		0.78		–2.99		.003	
**Area of work (reference group: operating rooms)**									
	Emergency department	–1.38 (–2.89 to 0.05)		0.75		–1.85		.07	
	Intensive care unit	0.55 (–1.06 to 2.2)		0.83		0.66		.51	
	Other inpatient services	–1.96 (–3.27 to –0.73)		0.65		–3.04		.002	
	Outpatient services	–1.18 (–2.95 to 0.49)		0.87		–1.36		.18	
**Specialty (reference group: nursing)**									
	Emergency medicine	–1.47 (–3.61 to 0.49)		1.04		–1.41		.16	
	Family medicine	–0.76 (–2.8 to 1.27)		1.03		–0.73		.46	
	Surgery	–2.25 (–4.42 to –0.25)		1.05		–2.14		.03	
	Anesthesiology	–1.14 (–2.41 to 0.09)		0.63		–1.80		.07	
	Gynecology	0.83 (–0.62 to 2.34)		0.75		1.11		.27	
**Years in practice (reference group: 16-20)**									
	<1	–1.25 (–3.57 to 1.12)		1.18		–1.06		.29	
	1-5	1.33 (–0.79 to 3.61)		1.11		1.21		.23	
	6-10	–0.93 (–3.02 to 1.2)		1.06		–0.88		.38	
	11-15	–0.12 (–2.12 to 1.95)		1.02		–0.12		.91	
**Type of hospital (reference group: public)**									
	Private	–0.21 (–1.65 to 1.19)		0.71		–0.29		.77	
	(Intercept)	11.30 (6.35 to 16.86)		2.65		4.27		<.001	

^a^OR: odds ratio.

^b^EHR: electronic health record.

We realize that the nurses and physicians working in hospitals that implemented various interventions regarding the use of technology at the workplace reveal lower odds of having any symptom of burnout compared to those working in hospitals that did not implement such interventions. Hospital preparedness was also associated with a reduction in burnout symptoms (OR –0.23, 95% CI –0.38 to –0.09; *P*=.002). Regarding the level of concern about COVID-19, we identified that the more the clinicians are concerned about the pandemic situation, the higher the odds of burnout (OR 0.29, 95% CI 0.11-0.48; *P*=.002). We did not find a significant association between the odds of having at least one symptom of burnout and the awareness about EHR features, use of technology solutions, and respondents’ professional efficacy.

Among the control variables, physicians (as a role), other inpatient services (as an area of work), and surgery (as a specialty) exhibited significantly lower burnout symptoms. Among the work areas, health care workers in other inpatient centers (eg, services delivered in rehabilitation centers, psychiatric departments, or addiction treatment centers) exhibited lower burnout.

We asked respondents about the factors that cause stress in their work in general prior to the pandemic. Almost one-fifth of the participants listed “insufficient compensation” or “family responsibilities” as the leading cause of stress. Almost one-fourth of the participants listed “workload” and almost one-fifth listed “health risks” as the second most important stressors. During the pandemic, the clinicians’ workload increased, and the concerns over contracting the virus or transmitting it to family members amplified. Almost one-fifth of the participants reported that they have work on average about 20 hours per week during the pandemic compared to the before the pandemic. Almost one-fourth of the participants reported that they have to work over the weekends more often during the pandemic compared to the time before it. Almost half of the participants reported an increase in the number of nights that they needed to be on call per week during the pandemic.

**Figure 4:**
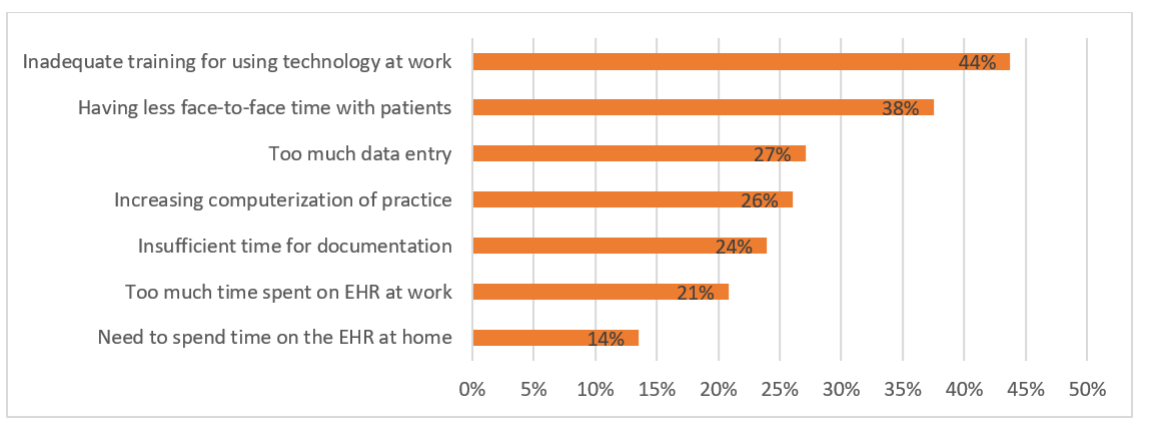
Leading causes of EHR-related stress during the COVID-19 pandemic. EHR: electronic health record.

We were also interested in understanding the EHR-related causes of stress during the pandemic. Figure 4 shows several EHR-related stress factors during the pandemic. During the pandemic, an extension was made to EHRs to register and keep track of COVID-19 tests administered and to send the results electronically to a national online repository. For the tests with positive results, the record of hospitalization or death was populated in the system. Respondents strongly agreed that the most important leading cause of EHR-related stress is inadequate training for using technology, followed by having less face-to-face time with patients, too much time spent on data entry, and increasing computerization at work. Since during the pandemic clinicians’ shifts changed and they had an additional workload with an unclear use policy, the likelihood of creating errors through using EHRs increased. Since the implementation of EHRs is still at an early stage, respondents mentioned inadequate training for using EHR features. This training issue, coupled with other key issues such as having less face-to-face time with patients (due to social distancing) and involving too much data entry (COVID-19 test results), caused more stress during the pandemic compared to normal times since the workload increased and job pressure was higher. The situational factors (due to the pandemic) exacerbated stress associated with EHR use in hospitals.

**Figure 5:**
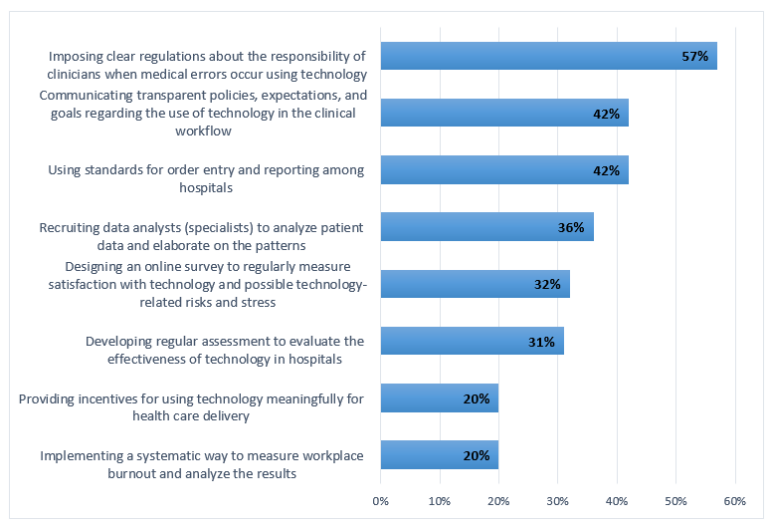
Effective hospital interventions during the COVID-19 pandemic.

Next, we asked participants about the effectiveness of implementing different interventions at the hospitals during the pandemic. Respondents believed that the hospitals’ most effective intervention was implementing clear regulations about the responsibility of clinicians when medical errors occur using technology. Clinicians are more prone to medical errors when they work under pressure and stress imposed by the pandemic. Therefore, the hospitals need to develop and implement proper regulations and guidelines, and communicate them clearly with the clinicians. Other effective interventions include imposing transparent policies, expectations, and goals regarding the use of technology in the clinical workflow and developing unified standards for order entry and reporting among hospitals (Figure 5).

**Figure 6:**
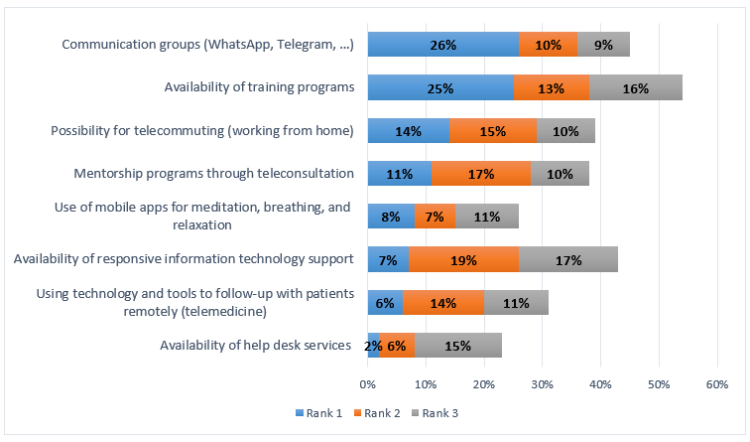
Effective technology solutions during the COVID-19 pandemic.

The most effective solutions that the respondents selected for addressing their burnout symptoms were using communication tools that facilitate group interactions (eg, WhatsApp and Telegram) and providing training programs for learning EHR systems. Respondents identified the second most effective solution as the availability of telecommuting (working from home), followed by participating in mentorship programs through teleconsultation (Figure 6). These findings show that the most prominent solution for reducing burnout among clinicians during the pandemic is facilitating the communication and information channels for them in a fast-paced environment to get the timely information they need to perform their activities. To practice social distancing, which is one of the main factors that can help reduce the transmission of COVID-19, the clinician needs to provide some of the care services remotely through telemedicine or teleconsultation. Our results show that special training to facilitate these activities for the clinician seems to be among the essential solutions to reduce clinicians’ burnout.

## Discussion

### Statement of Principal Findings

The COVID-19 pandemic imposed serious challenges to the health care systems of numerous countries. As this virus spreads worldwide, more research is needed to address clinicians’ burnout during the pandemic. This study aims to investigate burnout issues among physicians and nurses working in Iranian hospitals that directly provide care for patients suspected of or infected with COVID-19. In this study of 368 clinicians working in hospitals in Tehran, we found that EHR usability, technology-based hospital interventions, hospital preparedness, and level of concern about COVID-19 were significantly associated with workplace burnout. This finding is consistent with previous studies suggesting poor EHR usability will lead to clinicians’ dissatisfaction and, in turn, burnout [36]. EHRs have also been found to be a useful tool to support outbreak management [16]. Clinicians who believe that EHRs are not unnecessarily complicated and that they can have the support of technical personnel to use the system are less likely to experience burnout symptoms. Moreover, clinicians who consider that the various functions in EHRs are well integrated and that they can learn the features quickly are less prone to exhibit burnout symptoms. Given the negative association between EHR usability and burnout, hospitals need to improve EHR user-friendliness and convenience to reduce clinician burnout. Based on this study’s findings, we recommend that hospitals measure EHR usability and burnout among their clinicians in a regular and systematic way. Those clinicians who reported higher burnout symptoms could be the subject of a focus group to determine what changes are required by hospitals in implementing EHRs (for instance, availability of EHR-related training or technical support).

### Interpretation Within the Context of the Wider Literature

This study shows that the more caregivers are familiar with the EHR systems and feel confident in their ability to use them, the less they feel burnout symptoms. Interestingly in our sample, the most effective solution for reducing burnout symptoms was providing a training program for EHR use. Our results are in line with recent studies on EHR-related perceptions of workload that suggest individualized EHR training can improve the knowledge of EHR tools and satisfaction [37,38]. Other studies indicated that a considerable amount of time that the care providers need to spend recording information in the EHR system and data entry was the essential contributor to burnout [18]. However, our study reveals that the respondents are less worried about the data entry and their time using the system either at work or at home. They feel that more useful education and training programs are required to improve their symptoms of burnout. We also call attention to other important solutions to address burnout, such as easily accessible mentorship programs through teleconsultation and the availability of responsive IT support. Advanced EHR education through highly interactive personalized mentorship and hands-on workshops can help care providers alleviate workplace burnout and improve care quality [39,40]. EHR mentorship programs may improve the care providers’ self-efficacy, and the availability of 24-hour IT support provides the peace of mind that help is always accessible for them. This solution will help them combat burnout symptoms and psychological stress.

Our findings suggest that technology-related hospital interventions can decrease burnout among clinicians. We highlight several potential interventions to reduce the odds of burnout. Hospitals need to develop shared standards for data entry and exchanging health information among hospitals [41]. This intervention plays a significant role in the pandemic since prompt information sharing between hospitals and other health care organizations is required. Hospitals can also recruit business intelligence [38] specialists to analyze patient data, extract patterns, and illuminate risk factors and trends. Using reporting and analytics tools during the outbreak, data analysts can help hospitals devise better strategies by analyzing patient data from different perspectives (eg, location, symptoms, and contact with COVID-19–positive patients). Another possible intervention by hospitals could be using an online survey to regularly measure satisfaction with the technology used in their clinical setting. This approach can help hospitals identify potential technology-related risks and stress, reducing the likelihood of clinician burnout. Furthermore, hospitals can frequently evaluate the effectiveness of current technology. This approach provides hospitals with a better assessment of technology from the users’ perspectives. For instance, during the pandemic, hospitals may recognize the need to implement multiple COVID-19–specific tools (eg, an EHR-integrated patient self-triage and self-scheduling tool to manage COVID-19) [8].

### Implications for Policy, Practice, and Research

Based on the findings, a significant hospital intervention could be applying a systematic method for measuring workplace burnout and analyzing the results. Clinician burnout surveys could be conducted frequently. However, the findings and feedback could remain uninvestigated, and it will be unclear how burnout affects health care professionals’ productivity [42]. For instance, during the COVID-19 pandemic, clinicians may encounter new stressors that might not have been recognized previously [7]. Discovering stressors and analyzing the source of stress (eg, technology-related) can be useful for a rapid response to clinicians’ burnout during the COVID-19 outbreak.

Hospitals should establish transparent policies, expectations, and goals regarding the use of technology-based tools in the clinical workflow. For instance, the primary purpose of EHR implementation, the extent to which EHR features should be used, and clear regulations and guidelines for using EHRs should be communicated to clinicians working in hospitals. Otherwise, the lack of clear use strategies regarding clinician–technology interactions can lead to confusion, stress, and burnout [43]. First, we recommend that hospitals clarify clinicians’ responsibility, accountability, and associated regulations when medical errors occur using technology (eg, EHRs) in the clinical setting. Second, hospitals should define an *EHR meaningful use program* to elucidate what features of EHRs are essential and why their use is mandatory and, moreover, what applications are advanced or voluntary. Third, policy makers can offer incentives to hospitals and clinicians for the meaningful use of technology-based tools for health care delivery. These guidelines and instructions not only set a direction for hospitals on how to successfully implement and use EHRs but also mitigate the likelihood of clinician burnout. We need to acknowledge that any new policies, instructions, and procedures could be sources of burnout at the beginning (during a pandemic in particular). The success factor is implementing the new strategies, guidelines, and processes based on a robust execution plan, enough resources, and transparent communications and collaborations among clinicians in hospitals.

Clinicians working in the COVID-19 ward are significantly worried about becoming infected or concerned about their family becoming infected. This high level of concern about the pandemic significantly raises their burnout symptoms. The findings also demonstrate that hospital preparedness can enable the efficiencies of the COVID-19 care management program by reducing clinician burnout symptoms. Burnout is a system problem [44], and hospitals need to pose rapid solutions to address it during the pandemic. Hospitals can mitigate the odds of burnout among physicians and nurses by providing useful pandemic training, protective equipment, and support programs. Pandemic training (eg, classes, brochures, and meetings) can affect clinician stress by disseminating helpful statistics, insights, and information about the epidemic [45]. Through protective equipment (eg, personal protective equipment, masks, gloves, and sanitizer), hospitals are able to decrease workforce anxiety by providing clinicians facing patients with COVID-19 with various tools to avoid infection [46]. Support programs (eg, online safety alerts and wellness guidance, digital access to benefits, and work from home practices) can reduce burnout by improving work flexibility and helping clinicians access EHRs and other technology across distances [47].

### Limitations

This study is subject to some limitations. First, due to data collection limitations, our research is conducted based on a small group of physicians and nurses facing COVID-19. Second, this study is conducted in a resource-limited country with different pandemic preparedness plans and technology infrastructure in the health care system. Therefore, caution should be exercised when generalizing the findings to other countries. Third, because this study was conducted in the critical episode of the epidemic in Iran, selection bias may have skewed our results if highly stressed clinicians or those with little stress decided not to participate. Fourth, we conducted data collection electronically; thus, clinicians who were more comfortable with computers or mobile devices were more likely to participate. Finally, due to the time of data collection, our findings may not be generalized to the onset or the time that COVID-19 is under control in Iran. We recommend that future studies extend our research in the same or other contexts by collecting more comprehensive data using different data collection strategies, considering additional informatics-based tools, and examining further epidemic factors at different times.

### Conclusions

Studying technology-related factors affecting clinicians’ burnout has attracted much attention in the health care setting. This study adds to the literature by examining how EHR usability, technology-based hospital interventions, hospital preparedness, and concern about COVID-19 are significantly associated with burnout among clinicians during the COVID-19 pandemic in a resource-limited country. The results show the critical role of transparent hospital policies, EHR implementation strategies, training needs, and hospital pandemic support programs in reducing burnout. Interestingly, transparency is identified as the most important intervention that hospitals should implement to address burnout symptoms. Transparency in regulations addressing medical errors using technology and transparency dealing with policies and expectations of clinical workflows were identified as critical factors to alleviate workplace burnout. Due to the critical effects of burnout on physicians, nurses, patients, and the health care system, hospitals need to design robust interventions to address stress generated by HIT. Through this process, hospitals can mitigate the clinician burden and improve quality of care and patient safety.

## Appendix

Multimedia Appendix 1 Survey questions.

Multimedia Appendix 2 Sensitivity analysis.

Multimedia Appendix 3 Results after controlling for a hospital effect.

## Abbreviations

EHR: electronic health record
HIT: health information technology
IT: information technology
