# Supplementary material for: Using Electronic Health Records to Mitigate Workplace Burnout Among Clinicians During the COVID-19 Pandemic: Field Study in Iran
Source: JMIR Med Inform. 2021 Jun 3;9(6):e28497. doi: 10.2196/28497 (PMC8176947; doi:10.2196/28497)
Supplement: Multimedia Appendix 1 [file medinform_v9i6e28497_app1.docx]

**Appendix 1: Survey Questions**

Variable 1: Burnout prevalence

Select one from the following options:

Which of the following statements best reflects how you feel about your work?

1. I enjoy my work. I have no symptoms of burnout
2. I am under stress and don’t always have as much energy as I did, but I don’t feel burned out
3. I am definitely burning out and have one or more symptoms of burnout, e.g., emotional exhaustion
4. The symptoms of burnout I am experiencing won’t go away. I think about work frustrations a lot
5. I feel completely burned out. I am at the point where I may need to seek help
6. I am completely burned out, and I am getting help.

Variable 2: Please select the EHR features that are available and operational at your workplace:

Awareness of EHR features (Yes/ No):

Access to digital records (such as clinical notes, diagnoses, medication lists, vital signs, allergies, immunizations)

- View electronic clinical notes electronically
- View laboratory results electronically
- View imaging results electronically
- View immunization records
- View Allergies

Use of clinical charts

- - - Collect patient demographics electronically
    - List patient problems electronically
    - List patient medications electronically
    - Create medical history and follow-up notes

Use of retrieving diagnosis

- - - Send prescriptions electronically to a pharmacy
    - Send medical information securely to other healthcare professionals
    - Order laboratory tests electronically
    - Order radiology tests electronically
    - Highlight out-of-range laboratory results
    - Warn of drug interactions and contraindications
    - Receive reminders for guideline-based interventions
    - Receive reminders for preventive screening tests
    - Receive reminders for screens of chronic disease management
    - Sending Electronic referrals
    - Sending Electronic clinical messaging to patients
    - Remote access to EHR

Use of administrative systems :

-Personal human resources (payroll, benefits, training)

-Preregister systems (communication of some or all of the following item before patients arrive at the hospital: instructions, admission forms, patient safety, allergy, and comorbidity checklists, procedure preparation checklists, facility maps, and information packets, and letters from practice administrators and patients’ physicians)

Variable 3: EHR System Usability

*Think about the current electronic health record (EHR) you use most at work and indicate your response to the following statements.* (1- Strongly Disagree… 5-Strongly Agree)

1. I like to use EHR system.
2. I find the EHR system unnecessarily complex.
3. I think EHR is easy to use.
4. I think that I would need the support of technical personnel to use EHR better.
5. I find the various functions in EHR are well integrated.
6. I think there is too much inconsistency in EHR.
7. I would imagine that most people (in my specialty) would learn to use EHR quickly.
8. I find EHR very cumbersome to use.
9. I feel very confident in using EHR.
10. I needed to learn a lot of things before I could get going with EHR.
11. EHR improves communication among the physicians and staff in my practice

Variable 4: Concern about COVID-19 (1- Strongly Disagree… 5-Strongly Agree)

1. I feel more burnout now as compared with before the crisis of COVID
2. I am worried about becoming infected
3. I am worried about my family becoming infected
4. I am worried about this going for too long

Variable 5: Use of Technology Solutions (Current experience during the pandemic)

During the Pandemic, to what extent you use technology solutions for coping with work-related stress. Please rank the three most important items you have frequently used during the COVID-19 pandemic:

-Availability of training programs

-Availability of responsive information technology support

-Possibility for telecommuting (working from home)

-Using technology and tools to follow-up with patients remotely (telemedicine)

-Use of mobile apps for meditation, breathing, and relaxation

-Availability of helpdesk services

-Mentorship programs through teleconsultation

-Communication groups (what’s app, telegram, …)

-Others (please specify:___)

Variable 6: Hospital interventions during the Covid19 pandemic

To what extent you agree with the following items regarding the use of technology at your workplace? (1- Strongly Disagree… 5-Strongly Agree)

The hospital where I am working:

- Uses standards for order entry and reporting among hospitals

- Uses data analysts (specialists) to analyze patient data and elaborate on the patterns

- Uses an online survey to regularly measure satisfaction with technology and possible technology-related risks and stress

- Uses regular assessment to evaluate the effectiveness of technology in hospitals

- Uses a systematic way to measure workplace burnout and analyze the results

- Uses transparent policies, expectations, and goals regarding the use of technology in the clinical workflow

- Uses clear regulations about the responsibility of clinicians when medical errors occur using technology

- Provides incentives for using technology meaningfully for healthcare delivery

Variable 7: Pandemic preparedness: (1-Not helpful at all… 5- very helpful)

- - - - Please indicate to what extent your pandemic training (e.g., class, brochures, meeting) was helpful to control the health crisis?
      - Please indicate to what extent your hospital equipment (Personal Protective Equipment (PPE), Masks, Gloves, Sanitizer, etc.) was helpful to control the health crisis?
      - Please indicate to what extent your hospital support (e.g., online safety & wellness guidance, digital access to benefits, work from home practices) was helpful to control the health crisis?
      - To what extent did the hospital do everything possible to prioritize the safety of the employees?
      - To what extent did the hospital understand what they can do to improve the resilience of healthcare workers?

Variable 8: Profesional efficacy:

To what extent you agree with the following items regarding your professional efficacy during the pandemic? (1- Strongly Disagree… 5-Strongly Agree)

I feel confident I can keep myself healthy while doing my job during the pandemic

I feel confident in my ability to contribute during the pandemic

I feel confident in my knowledge to manage this health crisis

I feel confident in my professional spirit to handle this health crisis

Variable 9: General workplace stress

Which of the following items generally cause stress at the workplace: (Please rank the three most important stressors (1 being most important)

- Family responsibilities

- Time pressure

- Chaotic environment

- Unfavorable organizational culture (Conflict of values)

- Relationships with colleagues

- Lack of control (autonomy) over my work

- Insufficient compensation

-Workload

-Health risks

-Others (please specify:____)

Variable 10: EHR-related stress measures during the Covid19 pandemic

To what extent the following items cause stress for you during the Covid19 Pandemic? (1- Strongly Disagree… 5-Strongly Agree)

- Increasing computerization of practice

-Too much time spent on EHR at work

-Need to spend time on the EHR at home

- Insufficient time for documentation

- Too much data entry

-Having less face-to-face time with patients

- Inadequate technology-related training for using technology at work

-Others (please specify:….)

Participant Characteristics

Age

1. < 35

2. 35-44

3. 45-54

4. 55-64

5. >=65

Gender

1-Male

2-Female

Marital status:

1- Married

2-Single

Role:1- Nurse

2- Physician

3-Physician assistant

4-Other (please enter)

Area of work

Please specify the area of your work in a healthcare organization:

1-Emergency Department

2-Intensive Care Unit

3-Other inpatient services

4-Outpatient services

5-Others (please specify:___)

Specialty:

1-Emergency medicine

2-Family medicine

3-Psychiatry

4-Surgery

5. Anesthesiology

6. Pediatric

7. Gynecology

8. Nurse

9-Other (please specify…….)

How long have you been practicing? (years)

1- Less than one

2- One to five

3- Six to 10

4- 11-15

5. 16-20

5- More than 20

On average, for how many hours a week did you work before the COVID-19 pandemic?

1- <10

2- 11-20

3- 21-40

4-41-60

5- 61-80

6- >80

On average, for how many hours a week did you work in the past three months (during the pandemic)?

1- <10

2- 11-20

3- 21-40

4- 41-60

5- 61-80

6 ->80

How often did you work on weekends before the COVID-19 pandemic?

1- Every week

2- Every two weeks

3- Every three weeks

4- Monthly

5- Never

How often did you work on weekends for the past three months (during the pandemic)?

1- Every week

2- Every two weeks

3- Every three weeks

4- Monthly

5- Never

Number of nights on call per week before the COVID-19 pandemic? -----

Number of nights on call per week for the past three months (during the pandemic)? -----

Number of Nurses in your team ----

Number of Physicians in your team ---

Healthcare setting (the type of hospital)

1-Public hospital

2-Private hospital

3- Academic medical center

4-Nonprofit hospital

Number of covid19 cases handled:___
