# Supplementary material for: Using Electronic Health Records to Mitigate Workplace Burnout Among Clinicians During the COVID-19 Pandemic: Field Study in Iran
Source: JMIR Med Inform. 2021 Jun 3;9(6):e28497. doi: 10.2196/28497 (PMC8176947; doi:10.2196/28497)
Supplement: Multimedia Appendix 2 [file medinform_v9i6e28497_app2.docx]

**Appendix 2 Sensitivity analysis.**

The estimate of the association between practice and EHR characteristics and burnout symptoms among respondents; sensitivity analysis using ordinal responses for the dependent variable burnout instead of dichotomized responses

|  | **OR** | **(95% CI)** | **Std. Error** | **t value** | **p value** |
| --- | --- | --- | --- | --- | --- |
| **Awareness of EHR features** | -0.06 | (-0.11 -- 0.01) | 0.03 | -2.43 | 0.015 |
| **EHR system usability** | -0.09 | (-0.13 -- -0.07) | 0.02 | -6.24 | 0.000 |
| **Concerned about COVID19** | 0.20 | (0.11 -- 0.29) | 0.05 | 4.26 | 0.000 |
| **Use of technology solutions** | 0.01 | (-0.02 -- 0.05) | 0.02 | 0.71 | 0.474 |
| **Use of hospital technology interventions** | -0.14 | (-0.17 -- -0.09) | 0.02 | -6.33 | 0.000 |
| **Hospital preparedness** | -0.19 | (-0.27 -- -0.11) | 0.04 | -4.51 | 0.000 |
| **Professional efficacy** | 0.04 | (-0.02 – 0.10) | 0.03 | 1.14 | 0.254 |
| **Burnout** |  |  |  |  |  |
| 1\|2 | -7.00 |  | 1.15 | -6.06 | 0.000 |
| 2\|3 | -4.90 |  | 1.13 | -4.35 | 0.000 |
| 3\|4 | -3.39 |  | 1.12 | -3.04 | 0.002 |
| 4\|5 | -2.61 |  | 1.11 | -2.35 | 0.019 |
| 5\|6 | -1.11 |  | 1.11 | -0.99 | 0.318 |

Sensitivity analysis

| Outcome variable | Number of Covid-19 patients visited | | |
| --- | --- | --- | --- |
| Burnout | Less than 100 | More than 100 | Total |
| No symptom | 203 | 35 | 238 |
| One or more Symptoms | 92 | 38 | 130 |
| Total | 295 | 73 | 368 |

Chi-squared value = 3.07; critical chi-square=3.841; p-value= 0.079
