# Supplementary material for: Using Electronic Health Records to Mitigate Workplace Burnout Among Clinicians During the COVID-19 Pandemic: Field Study in Iran
Source: JMIR Med Inform. 2021 Jun 3;9(6):e28497. doi: 10.2196/28497 (PMC8176947; doi:10.2196/28497)
Supplement: Multimedia Appendix 3 [file medinform_v9i6e28497_app3.docx]

**Appendix 3** Results after controlling for a hospital effect.

The Estimate of the Association between Demographic, Practice, and EHR Characteristics and 1 or More Symptoms of Burnout after controlling for a hospital effect

| **Variable** | **OR** | **(95% CI)** | **Std. Error** | **z-value** | **p-value** |
| --- | --- | --- | --- | --- | --- |
| **Awareness of EHR features** | 0.04 | (-0.06 -- 0.14) | 0.05 | 0.73 | 0.468 |
| **EHR system usability** | -0.17* | (-0.26 -- -0.09) | 0.04 | -4.24 | 0.000 |
| **Concerned about COVID-19** | 0.27* | (0.09 -- 0.47) | 0.10 | 2.86 | 0.004 |
| **Use of technology solutions** | -0.06 | (-0.15 -- 0.02) | 0.04 | -1.40 | 0.161 |
| **Use of hospital technology interventions** | -0.39* | (-0.53 -- -0.27) | 0.07 | -6.00 | 0.000 |
| **Hospital preparedness** | -0.24* | (-0.10 -- -0.40) | 0.08 | -3.13 | 0.002 |
| **Professional efficacy** | -0.06 | (-0.23 -- 0.12) | 0.09 | -0.66 | 0.507 |
| **Age** |  |  |  |  |  |
| Less than 35 | -0.19 | (-3.69 -- 3.12) | 1.71 | -0.11 | 0.908 |
| 35-44 | -2.40 | (-5.93 -- 0.64) | 1.65 | -1.45 | 0.148 |
| 45-54 | -1.22 | (-4.57 -- 1.78) | 1.59 | -0.77 | 0.442 |
| 55-64 | 1.57 | (-1.51 -- 4.82) | 1.57 | 0.99 | 0.319 |
| 65 or higher | ref |  |  |  |  |
| **Gender** |  |  |  |  |  |
| Female | ref |  |  |  |  |
| Male | -0.82 | (-2.08 -- 0.39) | 0.62 | -1.32 | 0.188 |
| **Marital status** |  |  |  |  |  |
| Single | ref |  |  |  |  |
| Married | -0.28 | (-1.34 -- 0.75) | 0.53 | -0.53 | 0.593 |
| **Role** |  |  |  |  |  |
| Nurse | -0.75 | (-2.09 -- 0.56) | 0.67 | -1.11 | 0.267 |
| Physician | -2.11* | (-3.77 -- -0.60) | 0.80 | -2.63 | 0.009 |
| Physician assistant | ref |  |  |  |  |
| **Area of work** |  |  |  |  |  |
| Emergency department | -1.41 | (-2.94 -- 0.04) | 0.76 | -1.86 | 0.063 |
| ICU | 0.74 | (-0.91 -- 2.44) | 0.85 | 0.87 | 0.383 |
| Other inpatient services | -1.96* | (-3.16 -- -0.58) | 0.65 | -2.80 | 0.005 |
| Outpatient services | -1.11 | (-2.09 -- 0.58) | 0.88 | -1.26 | 0.208 |
| Operating Rooms | ref |  |  |  |  |
| **Specialty** |  |  |  |  |  |
| Emergency medicine | -1.61 | (-3.76 -- 0.35) | 1.04 | -1.55 | 0.120 |
| Family medicine | -0.81 | (-2.90 -- 1.27) | 1.06 | -0.77 | 0.443 |
| Surgery | -2.50* | (-4.73 -- -0.46) | 1.08 | -2.32 | 0.020 |
| Anesthesiology | -1.22 | (-2.53 -- 0.02) | 0.64 | -1.91 | 0.056 |
| Gynecology | 0.71 | (-0.76 -- 2.24) | 0.76 | 0.93 | 0.351 |
| Nursing | ref |  |  |  |  |
| **Years in practice** |  |  |  |  |  |
| Less than one | -1.19 | (-3.55 -- 1.23) | 1.20 | -0.99 | 0.323 |
| 1 - 5 | 1.42 | (-0.74 -- 3.75) | 1.13 | 1.26 | 0.209 |
| 6 - 10 | -0.89 | (-3.01 -- 1.27) | 1.08 | -0.83 | 0.409 |
| 11 - 15 | -0.03 | (-2.08 – 2.08) | 1.05 | -0.03 | 0.977 |
| 16 - 20 | ref |  |  |  |  |
| **Hospital** |  |  |  |  |  |
| Hospital 1 | 0.06 | (-1.54 – 1.65) | 0.80 | 0.07 | 0.943 |
| Hospital 2 | 0.52 | (-0.61 – 1.66) | 0.58 | 0.90 | 0.370 |
| Hospital 3 | -0.06 | (-1.28 – 1.13) | 0.61 | -0.11 | 0.909 |
| Hospital 4 | 0.51 | (-0.58 – 1.62) | 0.56 | 0.91 | 0.365 |
| Hospital 5 | 0.84 | (-0.27 – 1.98) | 0.57 | 1.47 | 0.141 |
| Hospital 6 | ref |  |  |  |  |
| (Intercept) | 11.59* | (6.46 – 17.35) | 2.74 | 4.22 | 0.000 |
